# The efficacy and safety of ^125^I brachytherapy combined with pre-operative transarterial chemoembolization in patients with locally advanced head and neck cancer

**DOI:** 10.3389/fonc.2022.992399

**Published:** 2022-09-14

**Authors:** Menglong Zhang, Jian Zhang, Bijuan Hu, Liyin Huang, Shanjun Song, Haitao Zhu, Chao Chen, Cunkun Chu

**Affiliations:** ^1^ Ganzhou People’s Hospital, Ganzhou, China; ^2^ Liaocheng People’s Hospital, Liaocheng, China; ^3^ Shandong Provincial Hospital Affiliated to Shandong First Medical University, Jinan, China; ^4^ Library, Shandong First Medical University and Shandong Academy of Medical Sciences, Tai’an, China

**Keywords:** brachytherapy, chemoembolization, head and neck cancer, intervention, efficacy

## Abstract

**Objective:**

To evaluate the safety and effectiveness of Iodine-125 **(** ^125^I) brachytherapy combined with pre-operative transarterial chemoembolization in patients with locally advanced head and neck cancer.

**Methods:**

In this study, a total of thirty-seven individuals suffering from locally advanced head and neck cancer were involved. The patients were subjected to transarterial chemoembolization as well as implantation of ^125^I seeds under the guidance of CT and ultrasonography. Follow-up was conducted for 36 months to study the following parameters: the local control rate, survival rate, and clinical complications.

**Results:**

In total, thirty-six patients at the end of three months showed an objective response rate of 69.8% and disease control rate of 93.0%, respectively. The 1, 2, and 3-year cumulative overall survival rate was 89.2%, 73.0%, and 45.9%, respectively. The adverse events of the treatment included infection (n=1, Grade III), radiation brachial plexus injury (n=1, Grade III), leukopenia (n=1, Grade III), cerebrovascular embolism (n=1, Grade IV).

**Conclusion:**

The combination of ^125^I brachytherapy and pre-operative transarterial chemoembolization was safe and effective in patients with locally advanced head and neck cancer.

## Introduction

The current global health statistics list head and neck cancer (including lip, oral cavity, larynx, nasopharynx, oropharynx, hypopharynx, salivary glands, and thyroid) as the seventh highest prevailing cancer with an affected population of 1518,000 cases and a death number of 510,000 per year around the globe (1). The status of malignancies of the head and neck as a locally advanced disease makes them difficult to treat requiring a multidisciplinary approach that incorporates surgery, radiotherapy, and systematic therapy (2). Currently, local recurrence is still the main cause of failure after surgery and radiotherapy, and there is no ideal method for salvage treatment after recurrence (3). It is necessary to explore non-surgical treatment methods to preserve organ functions in order to live a full and healthy life because the head and neck are necessary for speech and other important functions.

The minimally invasive nature of brachytherapy offers superior dosimetric advantages over conventional external beam techniques as it focuses the irradiation on the target site leading to much more precise dosimetric calculations as well as protecting the nearby tissues from radiation due to a sharp dose fall-off when the distance from the radiation source increases (4). Brachytherapy is one of the treatments recommended by the American Brachytherapy Society and the Head and Neck Working Group of the European Brachytherapy Group for head and neck cancers (5, 6). Evidence from previous studies shows that locally advanced head and neck cancer can be treated safely and effectively by the implantation of Iodine-125 (^125^I) radioactive seeds, a key element of brachytherapy, as a form of palliative salvage therapy (7–9). The long-term function of organs and cosmetic outcomes of the treatment is usually excellent. A meta-analysis has reported brachytherapy as a boost provides good clinical efficacy in terms of progression-free survival as compared to external beam radiation therapy (EBRT) in prostate cancer (10). A comparative study of high-dose-rate brachytherapy with volumetric modulated arc therapy in head and neck cancer showed comparable target coverage with a lower dose to normal tissues with brachytherapy (11).

Tumor hemorrhage is an important complication of cancer treatment, particularly in advanced head and neck cancer owing to spontaneous bleeding, iatrogenic vessel injury, pseudoaneurysm rupture or treatment effects (12). As head and neck malignancies may be located in deep tissues, and identification of their accurate position is extremely necessary for treatment. Therefore, traditional hemostatic methods, such as local compression, nasal packing, bandaging and suture, and drug hemostasis, are often ineffective. The increase in blood loss may lead to asphyxia and shock, which is very difficult to deal with clinically (12, 13). An alternative is presented by endovascular approaches with selective arterial embolization, which has a minimal impact on surrounding tissues. Transarterial embolization is performed by catheter insertion and has been reported to improve clinical outcomes significantly in head and neck cancer (14). Interventional chemotherapy with embolization has been used as adjuvant or palliative treatment, and although it cannot significantly increase the local drug concentration in tumors or reduce adverse reactions, it can block the arterial blood supply, promote tumor necrosis and shrinkage, as well as improve the curative effect, thereby improving prognosis (14, 15). In addition, interventional embolization before particle implantation can prevent acute bleeding during puncture (16). This study used arterial chemoembolization combined with particle implantation for locally advanced head and neck tumors four years ago. The outcomes of this combination therapy were retrospectively reported in this paper, together with an assessment of its effectiveness and any associated side effects.

## Patients and methods

### Patients

This retrospective investigation spanned from February 2018 to April 2022 and involved two hospitals covering thirty-seven individuals suffering from a malignant tumor of the head and neck. The main center provided most of the cases and only three cases were provided by the other hospital. The study protocol was approved by the Institutional ethical committee and all study procedures were consistent with the declaration of Helsinki. **Table 1** shows all the general information about the participants. The following were the criteria for the selection of cases in this study (1): patients in the age range of eighteen to eighty-five years with Karnovsky Performance Status (KPS) > 70 (2), the presence of head and neck squamous carcinoma pathologically diagnosed as grade III or grade IV with a lesion diameter< 7 cm and no metastasis (3), a possible needles pathway detected through the CT scan capable of being used for ^125^I brachytherapy-based implantation (4), an anticipated survival rate of more than 3 months (5), the rejection or ineligibility for external beam radiotherapy or salvage surgery, which may occur due to disease extent or systemic status. The exclusion criteria were as follows (1): acute organ failure (2), coagulation dysfunction (3), active infection (4), mental disorder, and (5) extensive necrosis/ulcer formation/skin rupture. An informed consent form for brachytherapy and interventional therapy had been signed by each patient.

**Table 1:** Basic characteristics of patients in this study.

Factor		Cases or number
Gender (male/female)		28/9
Age (years)		Median of 54 (25-81)
KPS (points)		Median of 80 (70-90)
Anatomic site		
Lip, oral cavity		7
Larynx		4
Nasopharynx		4
Oropharynx		5
Hypopharynx		2
Salivary glands		2
Thyroid		13
Tumor stage		
III		21
IV		16
Previous therapy		
surgery		18
radiotherapy		23
chemotherapy		17
Target therapy		7

### Transarterial chemoembolization

Seldinger technique was used to select an appropriate angiography catheter according to the vascular course, and a microcatheter was added for some patients. Selective arterial intubation was performed on different lesion sites, and drugs were injected after angiography to ensure no “dangerous anastomosis” and arteriovenous fistula. The chemotherapy regimen was docetaxel 60mg/m(2) with loplatin 30mg/m(2). The above drugs were diluted to 100 ml with normal saline and then slowly injected, respectively. After the infusion, embolization was performed with 300-500 um polyvinyl alcohol (PVA) particles.

### Iodine-125 brachytherapy

Before treatment, a spiral CT scan was performed, and tumor level data were input into a computer 3-D treatment planning system (TPS) to outline the tumor and the tumor target volume was simulated. The prescribed dose was set at 150Gy. The general principle of dose optimization was to achieve a clinical target volume (CTV) reaching prescription dose (PD) as far as possible while ensuring as low as possible exposure dose to normal tissues, and as few needles as possible. The specific limitations included (17) (1): determining the puncture trajectory to avoid vascularity, ventricles, and important structures (2). CTV100 (the CTV covered by more than 100% of PD) should be greater than 90% of the CTV (3). Organs at risk (OARs) were more than 1 cm away from the 100% PD isodose line. In this process, the radio-oncologist needs to adjust the needles and seeds repeatedly to meet those requirements. Dose-volume histogram (DVH) and real-time isodose contours were utilized for optimization. Patients were instructed to fast two hours before the operation and were given sedatives and lidocaine for local anesthesia before the operation, and their vital signs were monitored during the operation. Under the guidance of CT and B-ultrasound, the seed source was implanted into the tumor according to TPS. After the operation, the implant needle was pulled out and bandaged. Antibiotics and hemostatic drugs were routinely used three days after the operation to prevent postoperative infection and bleeding.

### Follow-up

The participants underwent a routine follow-up after a span of three months in the first year following ^125^I brachytherapy treatment, and 6 months in the later years. The patients underwent head and neck CT and enhanced MR, chest and abdomen multiphase CT, and laboratory examinations during each follow-up visit. The images obtained in the first and third months after ^125^I brachytherapy were utilized to analyze the response of the tumor to the treatment. The CT scan at the first follow-up was used for postoperative dose verification on the TPS and could be supplemented with seed implantation therapy when the dose was unevenly distributed and insufficient.

### Evaluations

The revised RECIST guideline (version: 1.1) was used as a reference to analyze the response of the tumor to the treatment in different aspects such as partial response (PR), complete response (CR), disease progression (PD), and stable disease (SD) at the first and second follow-up (18). The arterial phase enhancement lesion was detected using MRI and the selected lesion was identified as the target. This information was used to compute the objective response rate (ORR) and disease control rate (DCR) as follows:


$$ORR=\frac{CR+PR}{Total}\times 100\%,$$

$$DCR=\frac{CR+PR+SD}{Total}\times 100\%$$

The parameters, such as progression-free survival (PFS), the time between the initiation of treatment to the progress of the disease or death or even the time that the follow-up ends, and overall survival (OS), the duration of time survived from the initiation of treatment to either death or even the end of follow-up, were measured. The Radiation Therapy Oncology Group (RTOG) and the European Organization for Research and Treatment of Cancer (EORTC) were used to perform toxicity scoring of the radiation with reference to their toxicity criteria (19). The Common Terminology Criteria for Adverse Events v5.0 was utilized for analyzing the procedure-related side effects and complications associated with chemoembolization (20).

### Statistical analysis

SPSS 16.0 (SPSS Inc., Chicago, IL, USA) was utilized for statistical analysis of the data in the Windows operating system. The tumor response was calculated in percentage, whereas the Kaplan-Meier model was utilized to measure the survival rate. The number of different adverse events associated with the procedure was analyzed.

## Results

### Short-term efficacy

The locally assessable lesions in forty-three cases were treated with the help of a multidisciplinary approach combining arterial chemoembolization with the implantation of ^125^I seeds. Thirty-five lesions were successfully implanted at one time in line with TPS requirements, whereas eight lesions failed to meet the TPS requirements, so seeds were re-implanted one month after the first operation. The range of ^125^I seeds implanted in a single lesion was 9 ~ 45, with an average of 26 seeds. Local control rates were 95.3% and 93.0% at one and three months after treatment, respectively (**Table 2**).

**Table 2:** Short-term treatment effects of local lesions.

Efficacy	1 month (n, %)	3 months (n, %)
Complete response (CR)	2, 4.7%	11, 25.6%
Partial response (PR)	25, 58.1%	19, 44.2%
Stable disease (SD)	14, 32.6%	10, 23.3%
Progressive disease (PD)	2, 4.7%	3, 7.0%
Objective response rate (ORR)	28, 65.1%	30, 69.8%
Disease control rate (DCR)	41, 95.3%	40, 93.0%

### Long-term efficacy

**Figure 1:**
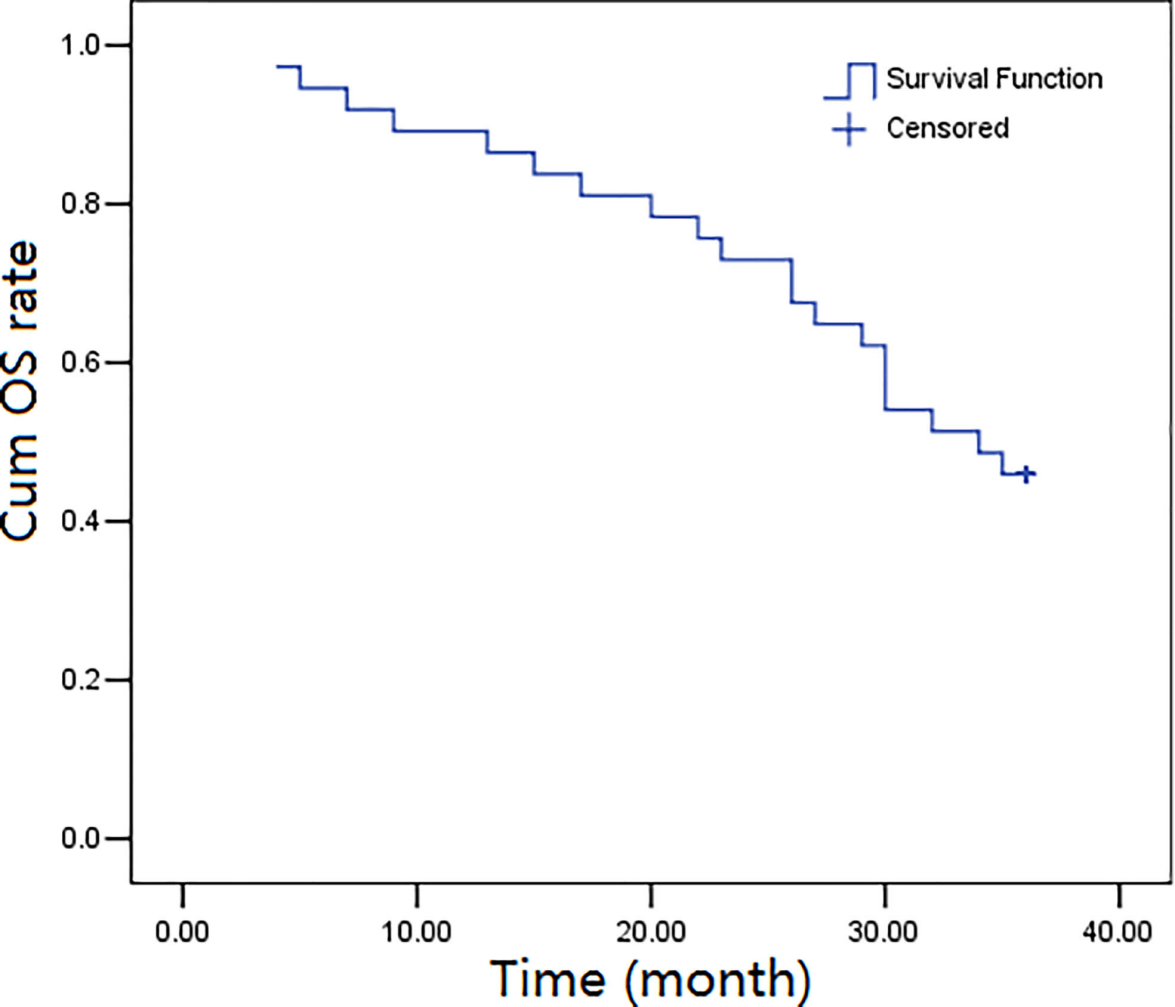
Kaplan-Meier overall survival (OS) curves for 37 cases.

**Figure 2:**
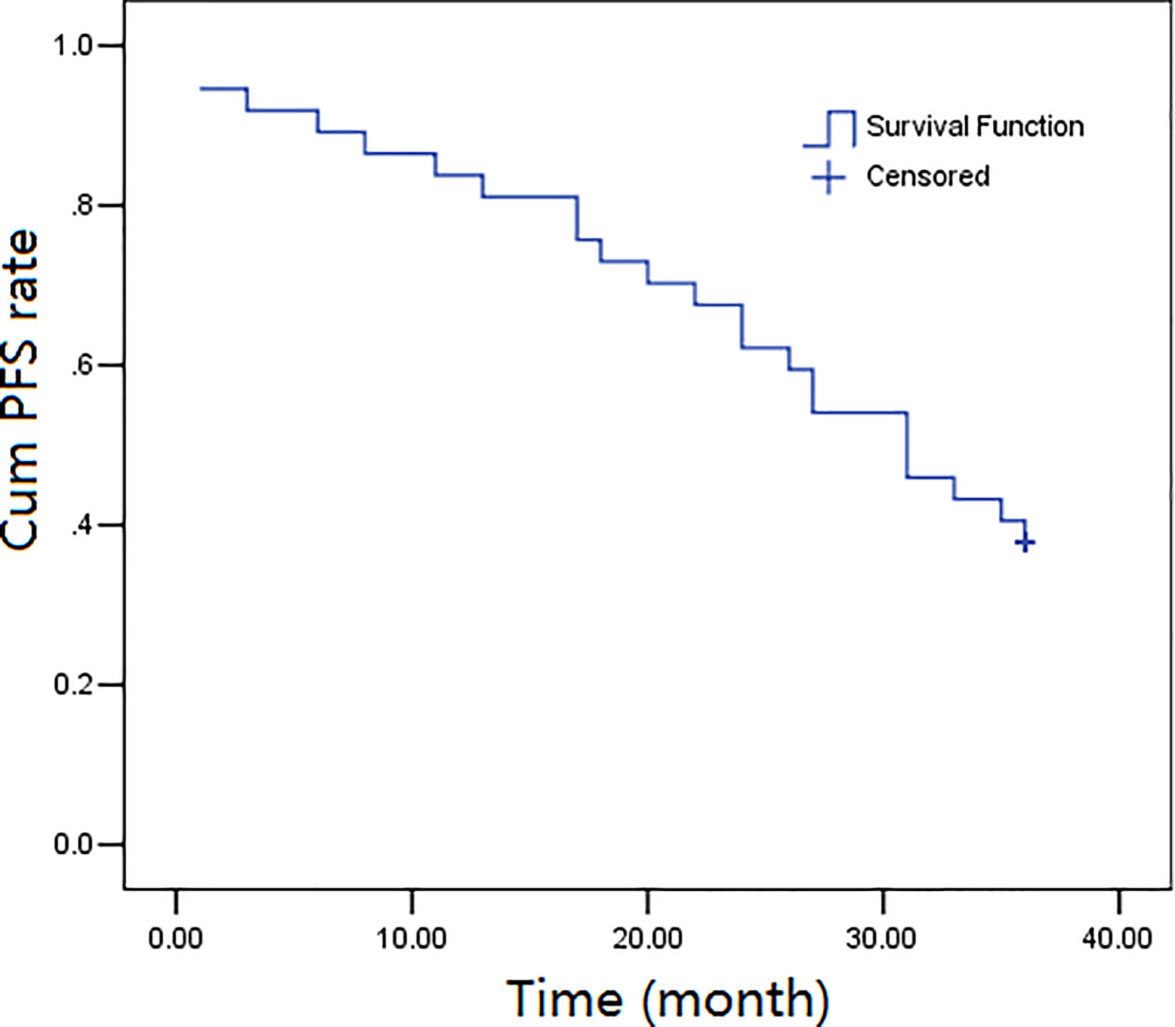
Kaplan-Meier progression-free survival (PFS) curves for 37 cases.

The three-year follow-up (52 months) was completed by all the thirty-seven participants, and the overall survival rates over 1, 2, and 3 years were calculated to be 89.2%, 73.0%, and 45.9%, respectively. The progression-free survival rates were calculated as 83.8%, 62.2%, and 37.8%, over 1, 2, and, 3 years, respectively. **Figures 1**, **2** depict the OS and PFS survival data.

### Adverse events

Adverse events occurred in thirty-five patients (94.6%), with grade 3 adverse events in four patients (10.8%), including one case each of puncture point pain, infection, puncture point pain with radioactive brachial plexus injury, and leukopenia. One patient (2.7%) had a grade 4 adverse event, namely cerebral artery embolism, but no grade 5 adverse events were detected (**Table 3**). Grade 3 and above adverse events were cured after symptomatic treatment, and treatment was discontinued without adverse reactions.

**Table 3:** Treatment-related adverse events.

Adverse events(n, %)		All	grade I	grade II	grade III	gradeIV
All		35	32	14	5	1
Puncture related*						
	Pain	31	26	4	2	
	Intraoperative Bleeding	0	–	–	0	
	Infection		–	5	1	
	Hematoma		13	2		
	Emphysema		4	2		
Arterial Chemoembolization*						
	Cerebrovascular Embolism					1
	Headache		2			
	Fall in Consciousness		3	1		
	Skin Itching, Numbness, Paresthesia		11	4		
	Vomiting		8	1		
	Diarrhea		2			
	Loss of appetite		16	2		
	Anemia		2			
	Leucopenia		10	6	1	
	Thrombocytopenia		1	1		
	Proteinuria			1		
	Fever		9	2		
	Alopecia		6	1		
Proximate Effect**						
	Skin		3			
	Hypodermis		1			
	Mucous Membrane		1	2		
	Salivary gland		3	1		
	Nerve				1	

*Classified and graded according to NCI-CTCAE 5.0 standard.

**Classified and graded according to RTOG/EORTC radiotoxicity classification criteria.

## Discussion

This research examined the effectiveness and adverse events of treating locally advanced head and neck malignancies using seed implantation in combination with arterial chemoembolization. The thirty-seven patients showed survival rates of 89.2%, 73.0%, and 45.9%, over 1, 2, and 3 years respectively with few serious complications. Adverse events of a higher grade occurred infrequently, with the incidence of grade 3 or higher events calculated to be 16.2%, and only one case of grade four event occurred. There was no serious intraoperative bleeding, which greatly improved the safety of the operation. This was one of the benefits of utilizing I-125 brachytherapy and chemoembolization.

The energy of radioactive seeds is low, and the dose reduces rapidly with distance, which makes it easy to confine the high-dose area to the target lesion, thereby enhancing the anti-tumor effect and minimizing the effects on normal tissues (21). Theoretically, radioactive seed implantation is appropriate for a solitary solid lesion. Since the blood supply arteries for head and neck tumors are relatively fixed, selective intubation can be easily put in place. Interventional local area chemotherapy can enable targeted drug delivery in large doses and high concentrations to lesions, greatly reducing the binding rate of drugs and plasma proteins, and considerably increasing the drug concentration of the lesion. It can suppress tumor cell proliferation, reduce tumor necrosis, cause apoptosis in a significant number of tumor cells and reduce the toxicity and side effects of systemic chemotherapy (22). The embolization of the supplying artery with a gelatin sponge can cause ischemic necrosis and shrinkage in the tumor (23, 24). At the same time, artery embolization before operation can reduce the bleeding caused by injury to the artery during puncture, and reduces the risk of operation.

Brachytherapy is a technique that is considered advantageous for most lip cancers as this kind of therapy helps to retain the salivary glands and other muscular functions (25). Furthermore, it is beneficial to treat most lip cancers (>90%) using brachytherapy as it offers treatment at all stages with a 90% to 95% local control rate as well as providing function retention of the organs and reducing the amount of scarring as a result of the operation. Brachytherapy is an advantageous technique for post-operative treatments in cases of tongue carcinoma when the organ to be treated shows signs of relapse due to risk factors such as perineural involvement, lymphovascular space invasion, and close margins. In such cases, external radiotherapy could cause more damage (xerostomia) and brachytherapy is a much more viable solution (26, 27). The implementation of the correct procedure of implantation of seeds leads to a significantly low and acceptable risk of developing serious complications (soft-tissue or bone necrosis) (<5%) (4).

Despite its numerous advantages brachytherapy is still difficult to attain complete success in the head and neck region as these two regions contain or are adjacent to many important organs (e.g. bones, sinuses, eyes, and major vessels), which may complicate the seed implantation process and lead to a dangerous situation. The practical implementation of the procedure is also difficult to achieve as the clinician’s errors in seed placement may cause more harm. Therefore, it is important to design a detailed preplan to minimize radioactive- and needle-associated damage to normal cells as well as perfectly implement the virtual plan in practice. Although techniques such as ultrasound, CT, MRI, and navigation have helped with reducing human error by guiding needle insertion and seed placement (28, 29), problems still occur. The human error and the limitations of the guiding technologies can be overcome by applying 3D printing templates (30, 31).

The timing of non-platinum-based chemotherapy and radiotherapy was examined in a study by UKHAN1 (32) to determine its impact on the effectiveness of the treatment. The study sought to ascertain whether the clinical results were affected by administering chemotherapy simultaneously with radiotherapy or as maintenance therapy, or both. The results depicted the advantage of two courses of concurrent non-platinum chemoradiotherapy treatment according to the reduced recurrence and mortality rate in patients who had not undergone previous surgery. Even after a long follow-up, there was no increase in the mortality and recurrence rate. From this study, it can be inferred that it may be best to perform arterial chemotherapy at the same time as the implantation of radioactive particles. In addition, a large-scale meta-analysis of randomized clinical trial and individual patient data comparing 16 different modalities for treatment of advanced head and neck cancer found that hyperfractionated radiotherapy with concomitant chemotherapy showed the best hazard ratio of 0.63. It is important to have randomized controlled trials of the present treatment modality to inform its comparative outcomes versus alternative clinical regimes (33). The present study is an observational study that has shown good safety and efficacy of I-125 brachytherapy combined with pre-operative transarterial chemoembolization. These data highlight the need for clinical trials in this domain.

The blood supplying arteries of tumors are generally abundant, and bleeding occurs easily when the tumor is punctured. Tumor hemorrhage can be complicated easily with infection, which not only increases the pain and economic burden of patients, but also spreads to the whole body and may lead to the death of patients with septic shock. Injury of large vessels causes large and rapid bleeding, leading to hemorrhagic shock and death. Heavy bleeding from the head and neck could lead to death by asphyxiation. Preoperative arterial embolization effectively prevented the occurrence of this situation (34). There were no bleeding events requiring medical intervention in this study.

Previous studies have shown that the incidence of short-term and long-term complications of head and neck tumor surgery with seed implantation is low (35, 36). The majority of adverse events associated with particle implantation therapy in this study were mild, only one patient progressed from preoperative grade II to III during the follow-up, and the patient had preoperative tumor invasion of brachial plexus accompanied by paresthesia and weak lifting power (grade 4 muscle strength). Three months after the operation, the tumor almost disappeared, but the neurological symptoms were aggravated, the paresthesia was more obvious than before, and the weakness was aggravated (muscle strength grade 3) while the nerve injury was upgraded from preoperative grade 2 to grade 3 (CTC 4.0 classification). However, the possibility of irreversible nerve injury before surgery and gradual aggravation after surgery due to tumor invasion cannot be completely ruled out.

In this study, the greatest risk of interventional surgery was cerebral infarction caused by internal carotid or vertebrobasilar artery system embolism. The only case of Grade 4 adverse event occurred due to this. This patient was a young woman, diagnosed with poorly differentiated nasopharyngeal squamous cell carcinoma. The nasopharyngeal tumor was uncontrolled after 3 months of radiotherapy so she was treated with I-125 brachytherapy by preoperative chemoembolization. After the operation, she was diagnosed with left eyeball abduction disorder, left central facial paralysis, decreased grip strength of left upper limb, lucidity, lethargy, and left limb paralysis and coma occurred the next day. The blood supply disturbance of the right middle cerebral artery was clinically diagnosed as caused by arterial embolism during interventional therapy. After dehydration, thrombolysis, vascular dilation, hormone therapy, microcirculation improvement, and intracranial pressure reduction, the patient returned to normal one week after the operation. To avoid this situation, angiography should be done before the injection of the embolic agent to make sure that there is no “dangerous anastomosis” and vasospasm before embolization. The embolization should be carried out after the super selection of target vessels, and complete embolization should not be forced. If blood flow is significantly slowed, the operation should be stopped. During embolization, the embolization should be strictly prevented from flowing back into the cervical or vertebrobasilar artery system (37, 38). In addition, pre and post-procedure oral hygiene management for prevention of complications is essential in brachytherapy for head and neck cancers (39). Furthermore, greater research regarding high-dose brachytherapy (HDR-BRT) is essential as increasing evidence shows its good clinical performance when used alone or as boost therapy (40). Moreover, as brachytherapy can be used as a definitive modality, boost modality and as salvage therapy, the clinical efficacy of the current protocol in these different investigations should be examined (41).

## Conclusion

In conclusion, ^125^I brachytherapy combined with preoperative arterial chemoembolization is a safe and feasible treatment for patients with locally advanced head and neck tumors. These patients achieved good rates of local control and overall survival with fewer serious adverse events. However, prospective randomized controlled trials are still needed to confirm the conclusions.

## Data availability statement

The original contributions presented in the study are included in the article/supplementary material. Further inquiries can be directed to the corresponding author.

## Ethics statement

The project was approved by the Ethics Committee of Ganzhou People’s Hospital, Ganzhou 341000, Jiangxi, China. The patients/participants provided their written informed consent to participate in this study.

## Author contributions

MZ conceptualized the research idea, carried out data analysis, led the paper writing, supervised the project, and edited the manuscript. JZ and BH performed the data analysis. LH, SS, HZ, CChe, and CChu were responsible for study design and data collection. All authors contributed to the article and approved the submitted version.

## Funding

This study was funded by the Science and Technology Program of Jiangxi Provincial Health and Welfare Committee (Grant No.: 202212514), Key Research and Development Program of Ganzhou City Science and Technology Bureau (Grant No.: 2020-275), Doctoral Research Initiation Project of Ganzhou People’s Hospital (Grant No.: Bsqd2020002), and Shandong Province Social Science Program Research Project (Grant No.: 20CTQJ07).

## Conflict of Interest

The authors declare that the research was conducted in the absence of any commercial or financial relationships that could be construed as a potential conflict of interest.

## Publisher’s Note

All claims expressed in this article are solely those of the authors and do not necessarily represent those of their affiliated organizations, or those of the publisher, the editors and the reviewers. Any product that may be evaluated in this article, or claim that may be made by its manufacturer, is not guaranteed or endorsed by the publisher.
